# Dual GIP/GLP-1 receptor agonist versus selective GLP-1 receptor agonist in patients undergoing primary total knee or hip arthroplasty

**DOI:** 10.1007/s00590-026-05000-9

**Published:** 2026-10-21

**Authors:** Spencer C. Davis, Shlok V. Patel, Shane Fletcher, John P. McLaughlin, Matthew E. Deren, Nicolas S. Piuzzi

**Affiliations:** 1 School of Medicine, Case Western Reserve University, Cleveland, USA; 2 Department of Orthopaedic Surgery, Cleveland Clinic, Cleveland, USA

**Keywords:** Tirzepatide, Semaglutide, GLP-1 receptor agonist, Total knee arthroplasty, Total hip arthroplasty, Periprosthetic joint infection

## Abstract

**Purpose:**

Tirzepatide is a dual glucose-dependent insulinotropic polypeptide (GIP) and glucagon-like peptide-1 (GLP-1) receptor agonist, whereas semaglutide is selective for the GLP-1 receptor. We compared post-operative outcomes after primary total knee or hip arthroplasty (TKA/THA) between patients prescribed tirzepatide and those prescribed semaglutide.

**Methods:**

A retrospective cohort study was performed using the TriNetX US Collaborative Network. Adults undergoing primary TKA or THA with at least two coded prescription fills for tirzepatide or semaglutide within six months of surgery were identified, and patients taking both agents were excluded. Cohorts were propensity score matched 1:1 (2,858 per group, all standardized differences below 0.05). Odds ratios (OR) with 95% confidence intervals (CI) were calculated at 90 days, 1 year, and 2 years.

**Results:**

Of 669,698 patients who underwent primary TKA or THA, 8,797 (1.3%) had ≥ 2 tirzepatide or semaglutide prescriptions. At 90 days and 1 year, no surgical, medical, or implant-related outcome differed significantly. At 2 years, tirzepatide exposure was associated with lower odds of mechanical complications (OR 0.64, 95% CI 0.42 to 0.96) and periprosthetic joint infection (PJI) (OR 0.64, 95% CI 0.41 to 0.99; *P* = .048). Reoperation did not differ significantly (OR 0.68, 95% CI 0.43 to 1.08).

**Conclusion:**

Tirzepatide and semaglutide prescriptions were associated with similar 90 day and 1 year outcomes, while tirzepatide was associated with lower mechanical complications and PJI at 2-years. These preliminary associations warrant further investigation in larger cohorts to improve precision and clarify their clinical relevance.

**Supplementary Information:**

The online version contains supplementary material available at https://doi.org/10.1007/s00590-026-05000-9.

## Introduction

Total knee and total hip arthroplasty (TKA/THA) are among the most frequently performed operations worldwide, and utilization has continued to rise across international health systems [1, 2]. In the United States they are among the most common and reliable orthopaedic procedures [3], and their effectiveness has prompted calls for arthroplasty to be recognized as an essential surgical service worldwide [4]. More than 7 million Americans currently live with a knee or hip replacement, and the annual volume of both procedures is projected to rise through 2030 [3, 5]. As incidence of these procedures increases, being able to identify modifiable patient factors that influence post-operative outcomes, such as periprosthetic joint infection and revision, becomes more important both clinically and economically [6, 7].

Glucagon-like peptide-1 receptor agonists (GLP-1RA) have become popular recently for treating type 2 diabetes and obesity [8, 9], and national prescribing has risen steeply over the past decade [10], which makes their perioperative management an increasingly common consideration in patients presenting for TKA or THA [11]. Pre-operative optimization in arthroplasty has traditionally used body mass index thresholds [12]. Because obesity and diabetes are established and modifiable risk factors for complications such as PJI and revision after surgery, these medications have received more attention as tools for pre-operative risk optimization as there is growing evidence that GLP-1RA exposure is associated with improved osteoarthritis symptoms and greater time to arthroplasty [13, 14]. Previous systematic reviews and institutional studies have generally reported comparable or lower complication rates among GLP-1RA users undergoing total joint arthroplasty, with lower or comparable rates of readmission, wound complications, and periprosthetic joint infection [15, 16].

Despite this, most previous literature has treated GLP-1RA as a single pharmacologic class, and direct comparisons between individual agents have not been well characterized. Tirzepatide is both a glucose-dependent insulinotropic polypeptide (GIP) and GLP-1 receptor agonist, while semaglutide is a selective GLP-1 receptor agonist [17, 18]. In recent clinical trial comparisons of these two drugs, tirzepatide has been shown to produce greater weight loss and glycemic improvement than semaglutide [17, 19]. Tirzepatide, which acts through dual GIP and GLP-1 receptor agonism, has been associated with reductions in liver fat content and visceral adipose tissue volume [20] and with lower circulating inflammatory markers [21], while GIP receptor activation has been associated with increased osteoblast-mediated bone formation and limited bone resorption [22]. Whether these pharmacologic differences translate into different post-operative outcomes after arthroplasty remains unclear. Accordingly, in this study, we compared associations with post-operative outcomes in patients taking tirzepatide vs. semaglutide prior to primary TKA or THA.

## Materials and methods

### Study design and data source

**Fig. 1:**
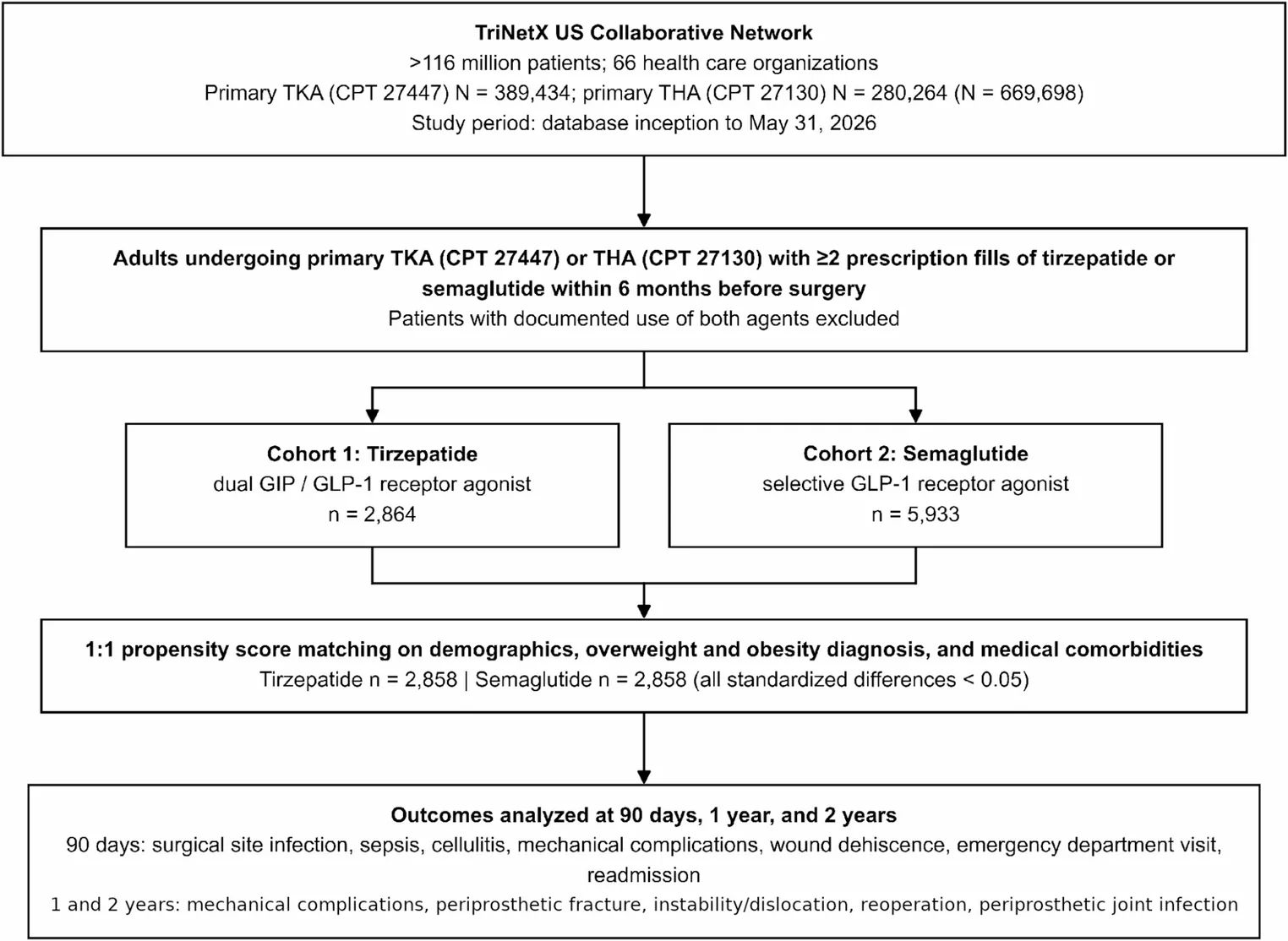
Strengthening the Reporting of Observational Studies in Epidemiology (STROBE) diagram of TriNetX cohort selection. TKA, total knee arthroplasty; THA, total hip arthroplasty; CPT, Current Procedural Terminology; GIP, glucose-dependent insulinotropic polypeptide; GLP-1, glucagon-like peptide-1

We performed a retrospective cohort study using the TriNetX Research Network (TriNetX, LLC, Cambridge, MA), a federated database of de-identified electronic health record data from over 60 participating health care organizations. The complete query criteria and outcome definitions used to construct each study cohort are provided in the Supplementary Material. Because all data in this platform are de-identified and contain no accessible protected health information (PHI), institutional review board approval and patient consent were not required for this study. Our analysis used the United States Collaborative Network, which contained a total of more than 116 million patients across 66 contributing healthcare organizations at the time of analysis. Study query was run on May 31, 2026. This study was designed and reported in accordance with the STROBE guidelines [23], and an overview of this study’s cohorts and methods is summarized in Fig. 1.

### Cohort definitions

Over the study period, 389,434 patients underwent primary TKA (CPT 27447) and 280,264 underwent primary THA (CPT 27130) in the network, for a total of 669,698 primary arthroplasties. Eligible patients for this study were adults who underwent primary TKA or THA and had at least two documented prescription fills for either tirzepatide (RxNorm 2601723) or semaglutide (RxNorm 1991302) within six months on or before the date of surgery. The two-fill requirement was used to strengthen our confidence that patients were taking the medication and reduce single-fill misclassification. Patients with any documented use of both medications were excluded.

### Covariates and matching

Cohorts were matched 1:1 using propensity score matching. Matching covariates included: age at index, sex, race, ethnicity, overweight and obesity (ICD-10 E66), hyperlipidemia (E78.5), type 2 diabetes mellitus (E11), long-term current use of insulin (Z79.4), chronic kidney disease (N18), nicotine dependence (F17), alcohol-related disorders (F10), and opioid-related disorders (F11). Covariate balance was assessed using standardized mean differences, with a value below 0.10 considered adequate balance.

### Outcomes

Outcomes were assessed at three time-points after surgery: 90 days, 1 year, and 2 years. Ninety-day outcomes included surgical site infection or infection following a procedure (ICD-10 T81.40 to T81.44 and T81.49), sepsis (A40, A41, R65.2), cellulitis of the lower extremity (L03.115, L03.116, L03.119), wound dehiscence or disruption (T81.3), mechanical complications, emergency department visits, and readmission. One- and two-year outcomes included: any mechanical complications, periprosthetic fracture (M97.0, M97.1), instability or dislocation (T84.02 group), reoperation (CPT 27134, 27137, 27138, 27090, 27091, 27486, 27487, 27488), and PJI (T84.50 to T84.59). Any mechanical complications represented any of the following: broken or dislocated prosthesis, instability, mechanical loosening, periprosthetic osteolysis, or articular bearing surface wear.

### Statistical analysis

Odds ratios (OR) with 95% confidence intervals (CI) were calculated within the TriNetX platform after 1:1 propensity matching, with statistical significance defined as *P* < .05. Our propensity-matched cohorts were analyzed across 90-day, 1-year, and 2-year timepoints. Outcomes with insufficient patient counts for analysis were not reported.

## Results

### Cohort characteristics

Of the 669,698 patients who underwent primary TKA or THA over the study period, 8,797 (1.3%) had at least two documented fills of tirzepatide or semaglutide within 6 months of surgery. Before propensity-score matching, 2,864 patients (0.4% of all primary TKA or THA) taking only tirzepatide and 5,933 (0.9%) taking only semaglutide met the inclusion criteria. These groups differed across several demographic and comorbidity variables, including age, race, ethnicity, overweight and obesity, type 2 diabetes mellitus, and hyperlipidemia (Table 1). After 1:1 propensity matching, 2,858 patients remained in each group, and all standardized differences were below 0.05, which confirmed adequate matching balance between cohorts (Table 1).

**Table 1:** Baseline characteristics before and after 1:1 propensity matching

Variable	Tirzepatide	Semaglutide	Std. Diff.	*P*-value
Before matching (Tirzepatide *N* = 2,864; Semaglutide *N* = 5,933)				
Age at index, mean ± SD	63.1 ± 8.49	63.8 ± 8.65	0.081	**< 0.001**
Female	1,899 (66.3)	3,873 (65.3)	0.022	0.342
White	2,311 (80.7)	4,553 (76.7)	0.097	**< 0.001**
Black or African American	364 (12.7)	973 (16.4)	0.105	**< 0.001**
Asian	22 (0.8)	66 (1.1)	0.036	0.128
Other / unknown race	175 (6.1)	341 (5.7)	NA	NA
Not Hispanic or Latino	2,389 (83.4)	4,705 (79.3)	0.106	**< 0.001**
Hispanic or Latino	103 (3.6)	267 (4.5)	0.046	**0.048**
Unknown ethnicity	372 (13.0)	961 (16.2)	0.091	**< 0.001**
Overweight and obesity	2,530 (88.3)	4,962 (83.6)	0.136	**< 0.001**
Type 2 diabetes mellitus	1,763 (61.6)	4,087 (68.9)	0.154	**< 0.001**
Hyperlipidemia	1,938 (67.7)	4,181 (70.5)	0.061	**0.007**
Long-term (current) insulin use	562 (19.6)	1,307 (22.0)	0.059	**0.010**
Chronic Kidney Disease	518 (18.1)	1,216 (20.5)	0.061	**0.008**
Nicotine dependence	460 (16.1)	966 (16.3)	0.006	0.793
Alcohol-related disorders	159 (5.6)	312 (5.3)	0.013	0.567
Opioid-related disorders	118 (4.1)	253 (4.3)	0.007	0.753
After 1:1 propensity matching (*N* = 2,858 per group)				
Age at index, mean ± SD	63.1 ± 8.48	63.3 ± 8.56	0.016	0.553
Female	1,894 (66.3)	1,906 (66.7)	0.009	0.737
White	2,311 (80.9)	2,323 (81.3)	0.011	0.685
Black or African American	364 (12.7)	371 (13.0)	0.007	0.782
Asian	22 (0.8)	14 (0.5)	0.035	0.181
Other / unknown race	169 (5.9)	159 (5.6)	NA	NA
Not Hispanic or Latino	2,383 (83.4)	2,374 (83.1)	0.008	0.750
Hispanic or Latino	103 (3.6)	111 (3.9)	0.015	0.577
Unknown ethnicity	372 (13.0)	373 (13.1)	0.001	0.969
Overweight and obesity	2,524 (88.3)	2,528 (88.5)	0.004	0.869
Type 2 diabetes mellitus	1,763 (61.7)	1,754 (61.4)	0.006	0.807
Hyperlipidemia	1,937 (67.8)	1,941 (67.9)	0.003	0.910
Long-term (current) insulin use	562 (19.7)	557 (19.5)	0.004	0.868
Chronic kidney disease	518 (18.1)	513 (17.9)	0.005	0.863
Nicotine dependence	460 (16.1)	442 (15.5)	0.017	0.514
Alcohol-related disorders	159 (5.6)	167 (5.8)	0.012	0.648
Opioid-related disorders	118 (4.1)	107 (3.7)	0.020	0.454

Values are n (%) unless otherwise specified. Std. Diff., standardized difference; a value below 0.10 indicates adequate balance after matching. Bold P-values denote statistical significance (*P*< .05). Race categories may not sum to the cohort total because more than one race may be recorded per patient

*SD* standard deviation

### Outcomes at 90 days

**Fig. 2:**
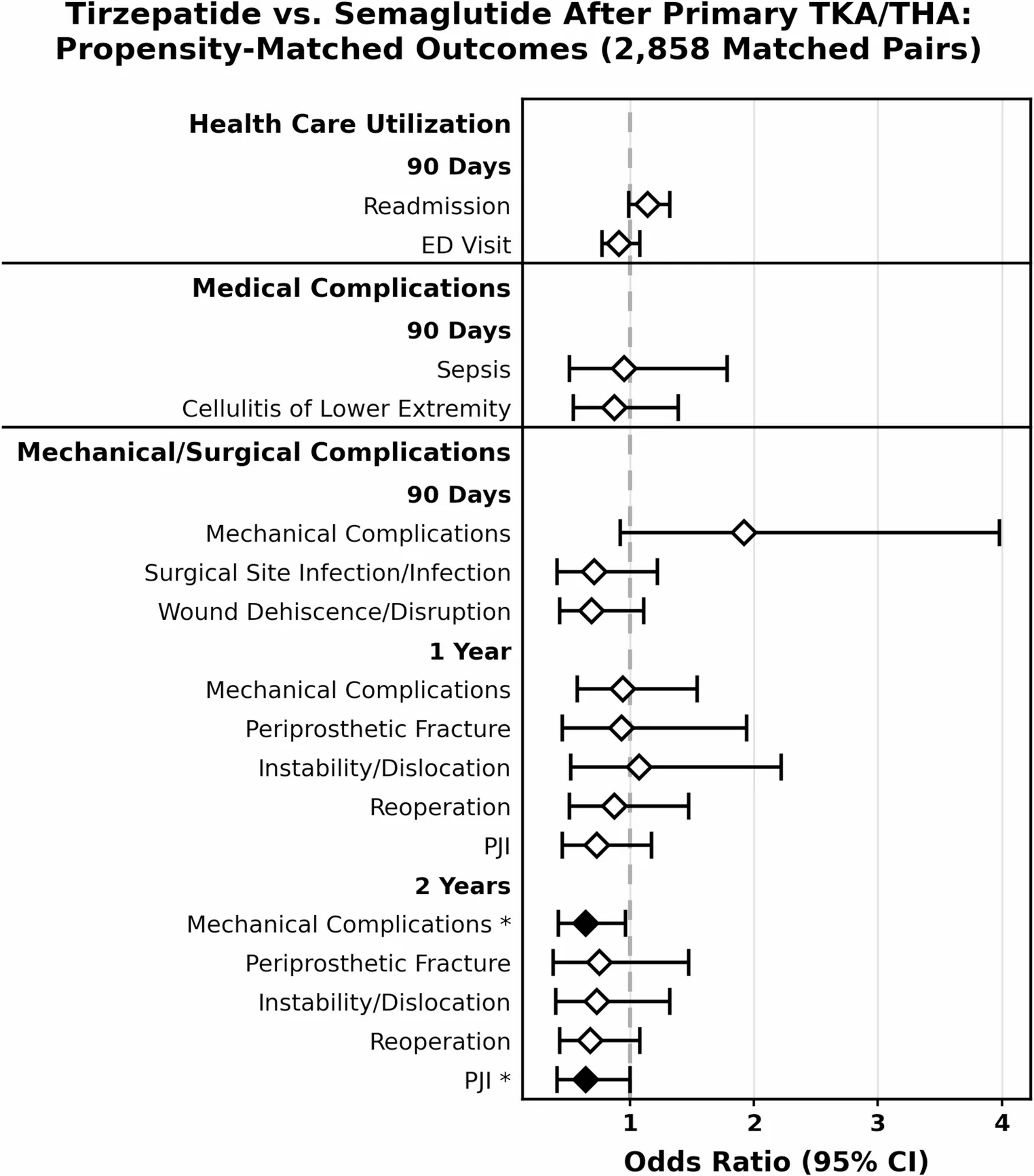
Propensity-matched odds ratios for tirzepatide versus semaglutide across all outcomes at 90 days, 1 year, and 2 years following primary TKA/THA. Odds ratios below 1.0 favor tirzepatide. Filled diamonds and asterisks denote *P* < .05. OR, odds ratio; CI, confidence interval; ED, emergency department; PJI, periprosthetic joint infection; TKA, total knee arthroplasty; THA, total hip arthroplasty

At 90 days, infection and wound-related outcomes were comparable between tirzepatide and semaglutide patients, including surgical site infection (OR 0.71, 95% CI 0.41 to 1.22), sepsis (OR 0.95, 95% CI 0.51 to 1.78), cellulitis (OR 0.87, 95% CI 0.54 to 1.39), and wound dehiscence or disruption (OR 0.69, 95% CI 0.43 to 1.11). Emergency department visits (OR 0.91, 95% CI 0.77 to 1.08), readmission (OR 1.14, 95% CI 0.99 to 1.32), and early mechanical complications (OR 1.92, 95% CI 0.92 to 3.98) did not differ significantly between cohorts (Table 2; Fig. 2).

**Table 2:** Post-operative outcomes after primary TKA/THA following 1:1 propensity matching

Outcome	Tirzepatide *n*	Semaglutide *n*	OR (95% CI)	*P*-value
90 days (*N* = 2,858 per group)				
Surgical site infection / infection following procedure	22	31	0.71 (0.41–1.22)	0.214
Sepsis	19	20	0.95 (0.51–1.78)	0.872
Cellulitis of lower extremity	33	38	0.87 (0.54–1.39)	0.550
Mechanical complications	21	11	1.92 (0.92–3.98)	0.076
Wound dehiscence / disruption	30	43	0.69 (0.43–1.11)	0.126
Emergency department visit	286	312	0.91 (0.77–1.08)	0.261
Readmission	454	406	1.14 (0.99–1.32)	0.076
1 year				
Mechanical complications	31	33	0.94 (0.57–1.54)	0.801
Periprosthetic fracture	14	15	0.93 (0.45–1.94)	0.852
Instability / dislocation	15	14	1.07 (0.52–2.22)	0.852
Reoperation	26	30	0.87 (0.51–1.47)	0.591
Periprosthetic joint infection	30	41	0.73 (0.45–1.17)	0.189
2 years				
Mechanical complications	38	59	**0.64 (0.42–0.96)**	**0.032**
Periprosthetic fracture	15	20	0.75 (0.38–1.47)	0.397
Instability / dislocation	19	26	0.73 (0.40–1.32)	0.295
Reoperation	30	44	0.68 (0.43–1.08)	0.101
Periprosthetic joint infection	33	51	**0.64 (0.41–0.999)**	**0.048**

OR < 1.0 favors tirzepatide. The matched cohort comprised 2,858 patients per group

*n*number of patients with the outcome in each cohortL *OR* odds ratio, *CI* confidence interval. *TKA* total knee arthroplasty, *THA* total hip arthroplasty, *PJI* periprosthetic joint infection

Bold rows denote statistical significance (*P *< .05)

### Outcomes at 1 year

At 1 year, all implant-related outcomes remained comparable between groups, with every confidence interval crossing one. These included any mechanical complication (OR 0.94, 95% CI 0.57 to 1.54), periprosthetic fracture (OR 0.93, 95% CI 0.45 to 1.94), instability or dislocation (OR 1.07, 95% CI 0.52 to 2.22), reoperation (OR 0.87, 95% CI 0.51 to 1.47), and PJI (OR 0.73, 95% CI 0.45 to 1.17) (Table 2; Fig. 2). 

### Outcomes at 2 years

At 2 years, tirzepatide use was associated with significantly lower odds of mechanical complications (OR 0.64, 95% CI 0.42 to 0.96) and PJI (OR 0.64, 95% CI 0.41 to 0.999; *P* = .048). Reoperation did not differ significantly (OR 0.68, 95% CI 0.43 to 1.08). Periprosthetic fracture (OR 0.75, 95% CI 0.38 to 1.47) and instability or dislocation (OR 0.73, 95% CI 0.40 to 1.32) also did not differ significantly (Table 2; Fig. 2).

## Discussion

Tirzepatide or semaglutide was documented in 8,797 (1.3%) of the 669,698 patients who underwent primary TKA or THA in this database. In this propensity-matched cohort analysis, tirzepatide and semaglutide use within 6 months prior to surgery were associated with comparable post-operative outcomes at 90-days and 1 year across medical, surgical, and implant-related outcomes. At 2 years follow-up, tirzepatide use was associated with significantly lower odds of mechanical complications and PJI, while reoperation did not differ significantly. These findings suggest a possible difference in longer-term outcomes between the two agents. The strength of evidence supporting these associations remains limited, and larger studies are needed to clarify their magnitude and consistency.

These findings build on literature that has primarily examined outcomes of GLP-1RA grouped as a single pharmacologic class [11, 24]. Prior systematic reviews and large database analyses have generally reported comparable or decreased complications among GLP-1RA users undergoing arthroplasty, including lower rates of readmission, wound complications, and PJI, and single-agent and mixed-class analyses have also found no increase in early complications [15, 16]. Our results extend this literature by directly comparing tirzepatide and semaglutide. No significant differences were observed in the assessed outcomes at 90 days or 1 year.

 At 2 years, tirzepatide use was associated with lower odds of mechanical complications and PJI, but both estimates were borderline (*P* = .032 and 0.048; upper confidence limits 0.96 and 0.99). These findings should be interpreted cautiously given the limited number of outcome events and the multiple comparisons performed. Several other mechanisms could plausibly contribute to the associations observed at 2 years. Tirzepatide has been shown to produce greater and longer lasting weight loss than semaglutide in comparative clinical trials [17, 19], and because obesity is among the most important modifiable risk factors for PJI and several mechanical complications [7], the greater weight reduction association with tirzepatide could plausibly contribute to the lower odds observed in this study. The additional GIP receptor activity of tirzepatide compared to semaglutide may also be relevant, as GIP receptors are expressed on osteoblasts and osteoclasts, where incretin signaling has been associated with increased bone formation and reduced bone resorption and could possibly strengthen implant fixation and preserve periprosthetic bone quality [22]. GLP-1 signaling has also been associated with anti-inflammatory effects that are consistent with the associations with lower odds of PJI found in our study [18].

These findings carry several clinical implications. The absence of significant differences in the assessed outcomes at 90 days and 1 year provides no clear basis for favoring either agent on the basis of early arthroplasty outcomes. However, the observed associations with lower odds of mechanical complications and PJI at 2 years provide preliminary evidence that agent selection could be relevant to longer-term implant outcomes in patients receiving incretin-based therapy. Larger comparative studies that account for treatment duration, dosing, and changes in weight and glycemic control would help clarify whether the observed associations reflect differences between the agents or the patients receiving them. Such evidence would help determine whether arthroplasty outcomes should be considered alongside established metabolic indications when selecting therapy.

This study has several limitations. Most importantly, medication exposure was defined from electronic health record coding, which does not confirm that medications were taken, and adherence to medications may correlate with metabolic control and other factors that could independently influence perioperative risk. We could not stratify by dosage, treatment duration, or indication, and we could not distinguish patients treated for diabetes from those treated for obesity. Overweight and obesity diagnosis (ICD-10 E66) was included as a matching covariate, but residual confounding by the degree and trajectory of previous weight change cannot be excluded. All outcomes were identified from coded diagnoses and procedures, which are subject to misclassification, and we could not distinguish ipsilateral from contralateral events. Patient event counts were limited for several implant-related outcomes, which reduced precision and prevented analysis of rarer outcomes. Multiple outcomes were assessed across three time points without adjustment for multiple comparisons, which increases the likelihood that at least one significant association could reflect chance. Finally, this was a retrospective database study and cannot establish causation.

## Conclusion

Among 5,716 propensity-matched patients who underwent primary TKA or THA, tirzepatide and semaglutide exposure were associated with comparable post-operative outcomes at 90-days and 1 year. At 2 years, tirzepatide exposure was associated with significantly lower odds of mechanical complications and periprosthetic joint infection, while reoperation did not differ significantly. These findings suggest a potential difference in longer-term outcomes between dual GIP/GLP-1 receptor agonism and selective GLP-1 receptor agonism. Larger prospective comparative studies that incorporate dispensing data, dosing, and weight trajectory are needed to determine whether the choice of incretin-based therapy is associated with clinically meaningful differences in longer-term arthroplasty outcomes.

## Supplementary Information

Supplementary Material 1.

## Data Availability

The aggregated data supporting the findings of this study were generated within the TriNetX Research Network and are available to investigators at institutions holding a TriNetX subscription. Patient-level data are not available to the authors, as the platform returns only aggregated counts and summary statistics derived from de-identified records.
