# Supplementary material for: Dual GIP/GLP-1 receptor agonist versus selective GLP-1 receptor agonist in patients undergoing primary total knee or hip arthroplasty
Source: Eur J Orthop Surg Traumatol. 2026 Oct 21;36(1):417. doi: 10.1007/s00590-026-05000-9 (PMC13687161; doi:10.1007/s00590-026-05000-9)
Supplement: Supplementary file 1 [file 590_2026_5000_MOESM1_ESM.docx]

**Supplementary Material**

**Tirzepatide versus Semaglutide TKA/THA**

**Compare Outcomes Analysis**

Created by TriNetX

**Introduction**

TriNetX is the global federated health research network providing access to electronic medical records (diagnoses, procedures, medications, laboratory values, genomic information) across large healthcare organizations (HCOs). This report was run on the set of HCOs grouped into a network called US Collaborative Network. This network included 66 HCO(s).

This report describes the Compare Outcomes Analyses generated by the TriNetX platform for the study titled Tirzepatide versus Semaglutide TKA/THA. The following cohorts were defined and analyzed: Cohort 1 (2,864 patients) named Tirzepatide TKA/THA 2x; Cohort 2 (5,933 patients) named Semaglutide TKA/THA 2x.

Eligible patients were adults who underwent primary TKA (CPT 27447) or THA (CPT 27130) and had at least two documented prescription fills for either tirzepatide or semaglutide within six months on or before the date of surgery. Patients with any documented use of both agents were excluded. The two cohorts were compared in a single 1:1 propensity-matched analysis.

**Methods**

The analysis process includes two main steps: 1) Defining the cohorts through query criteria; 2) Setting up and running the analysis. Setting up the analysis requires definitions for the index event, outcomes criteria, and the time frame. Compare outcomes supports four analyses: Measures of Association, Survival, Number of Instances and Lab result distribution. These analyses have additional options that are listed in the Outcomes Definitions and Analyses Specifications section below. Furthermore, characteristics of the cohorts that are balanced using propensity score matching are also included in the Propensity Score Matching section.

**Cohorts definition**

This section lists all terms used in the definitions of the cohorts.

**Query Criteria for Cohort 1 (query name: Tirzepatide TKA/THA 2x)**

This query was run on the network US Collaborative Network with 66 HCO(s) queried and 66 HCO(s) responded. A total of 42 HCO(s) responded with patients. The final cohort included 2,864 patients who matched the query criteria listed in the table below.

| **Group 1** | | | | | |
| --- | --- | --- | --- | --- | --- |
| **Group 1A TKA/THA** | | | | | |
|  | must have | any of | procedure | UMLS:CPT:27447 | Arthroplasty, knee, condyle and plateau; medial AND lateral compartments with or without patella resurfacing (total knee arthroplasty) |
|  |  |  | procedure | UMLS:CPT:27130 | Arthroplasty, acetabular and proximal femoral prosthetic replacement (total hip arthroplasty), with or without autograft or allograft |
|  | **date constraint** | The terms in this group occurred at any time | | | |
|  | **event relationship** | Any instance of Tirzepatide occurred within 6 months on or before any instance of TKA/THA | | | |
| **Group 1B Tirzepatide** | | | | | |
|  | must have | any of | medication | UMLS:RXNORM:2601723 | tirzepatide |
|  | cannot have |  | medication | UMLS:RXNORM:1991302 | semaglutide |
| **AND** | | | | | |
| **Group 2** | | | | | |
| A patient must have greater than or equal to 2 instances of these terms | | | | | |
| **Tirzepatide** | | | | | |
|  | must have | any of | medication | UMLS:RXNORM:2601723 | tirzepatide |
|  | **date constraint** | The terms in this group occurred at any time | | | |

**Query Criteria for Cohort 2 (query name: Semaglutide TKA/THA 2x)**

This query was run on the network US Collaborative Network with 66 HCO(s) queried and 66 HCO(s) responded. A total of 49 HCO(s) responded with patients. The final cohort included 5,933 patients who matched the query criteria listed in the table below.

| **Group 1** | | | | | |
| --- | --- | --- | --- | --- | --- |
| **Group 1A TKA/THA** | | | | | |
|  | must have | any of | procedure | UMLS:CPT:27130 | Arthroplasty, acetabular and proximal femoral prosthetic replacement (total hip arthroplasty), with or without autograft or allograft |
|  |  |  | procedure | UMLS:CPT:27447 | Arthroplasty, knee, condyle and plateau; medial AND lateral compartments with or without patella resurfacing (total knee arthroplasty) |
|  | **date constraint** | The terms in this group occurred at any time | | | |
|  | **event relationship** | Any instance of Semaglutide occurred within 6 months on or before any instance of TKA/THA | | | |
| **Group 1B Semaglutide** | | | | | |
|  | must have | any of | medication | UMLS:RXNORM:1991302 | semaglutide |
|  | cannot have |  | medication | UMLS:RXNORM:2601723 | tirzepatide |
| **AND** | | | | | |
| **Group 2** | | | | | |
| A patient must have greater than or equal to 2 instances of these terms | | | | | |
| **Semaglutide** | | | | | |
|  | must have | any of | medication | UMLS:RXNORM:1991302 | semaglutide |
|  | **date constraint** | The terms in this group occurred at any time | | | |

**Analysis Setup**

This section contains the Index Event and Time Window definitions and a list of selected outcomes and the analyses.

**Index Event & Time Window Definitions**

The index event defines the point in time when each patient in the cohort enters the analysis. To define an index event for the cohort, one or more criteria for the cohort must be selected. The index date for each patient within a cohort is the day on which the patient first met the selected criteria for the cohort.

As the index event defines the earliest time point after which outcomes are analyzed, the time window defines the duration during which outcomes are analyzed. The time window can start on the same day as the index event or at any specified time interval after the index event. The time window can end any time after the start date. Outcomes are defined as diagnoses, medications, procedures, or laboratory values that happened in the time window starting after the first occurrence of the index event.

**Time Window Used in this Analysis**

Outcomes were assessed at three time-points after surgery: 90 days, 1 year, and 2 years.

**Index Events Used in this Analysis**

Index events for the Compare Outcomes analysis were derived from the cohort definitions. Index events were defined separately for each cohort and were based on the criteria used in the original cohort definition.

Eligible patients for this study were adults who underwent primary TKA (CPT 27447) or THA (CPT 27130). The date of the index arthroplasty served as the index event for each patient.

**Analyses Specifications**

The Compare Outcomes Analytic supports four types of analyses: Measure of Association, Survival, Number of Instances, and Lab result distribution. The first three analyses support the "exclude patients with outcomes prior to the window" setting. This option can exclude patients from the analysis if they are not at risk for an outcome (e.g., if the outcome is a chronic disease). When "exclude patients with the outcome prior to the time window" is not checked, all patients in the cohort are included in the analysis, regardless of whether they had the outcome prior to the time window. When "exclude patients with the outcome prior to the time window" is checked, patients are excluded from the analysis if their record includes the outcome prior to the beginning of the time window. This selection will exclude all patients with the outcome prior to the index event. If the start of the time window for the analysis falls some days after the index event, patients will also be excluded if they have the outcome between the index event and the start of the time window.

**Measure of Association Analysis**

The Measure of Association Analysis calculates and compares the fraction of patients with the selected outcome. The output summary includes: Patients in each Cohort (count of patients meeting query criteria); Patients with Outcome in each Cohort (of the patients in the cohort, count of patients that had the outcome in the time window); and Risk (the fraction of patients in the cohort that have the outcome in the time window, i.e. Patients with Outcome / Patients in Cohort). In addition, Risk Difference (the difference in the risks in Cohort 1 and Cohort 2), Risk Ratio (the ratio of the risks in Cohort 1 and Cohort 2), and Odds Ratio (the ratio of the odds in Cohort 1 and Cohort 2). The bar chart shows the risk of the outcome for the both cohorts.

**Survival Analysis**

The Kaplan-Meier Analysis estimates probability of the outcome at a respective time interval (daily time interval is used in this analysis). In order to account for the patients who exited the cohort during the analysis period, and therefore should not be included in the analysis, censoring is applied. In this analysis, patients are removed from the analysis (censored) after the last fact in their record.

The output summary includes: Patients in each Cohort (count of patients meeting query criteria); Patients with Outcome (of the patients in the cohort, count of patients that had the outcome in the time window); Median Survival (the number of days when the survival drops below 50%; the "-" indicates that survival does not drop below 50% during the time window); and Survival Probability at End of Time Window (the % survival at the end of the time window). In addition, Log-Rank test, Hazard Ratio and test for Proportionality.

**Number of Instances Analysis**

The Number of Instances Analysis calculates how many times the outcome occurred in the time window. This analysis includes two additional settings: include patients with zero instances; the definition of an instance.

Selecting to exclude patients with zero instances will remove these patients from the calculations for mean number of instances, standard deviation, or median. The histogram showing the distribution of patients by number of instances will not contain a bar for zero. Alternatively, by selecting to include patients with zero instances, the mean, standard deviation, and median for number of instances will reflect these patients. The histogram will contain a bar for zero patients.

The definition of an instance affects how counts are analyzed. By selecting Date, each calendar date on which any of the terms selected in the outcome are recorded will represent one instance. For example, if the outcome is "Med A or Med B," and a patient has "Med A" on January 3, then both medications on January 4, then "Med B" on January 6, then that patient is considered to have three instances- January 3, January 4, and January 6. Note that if an outcome occurs across several dates (e.g. Visit: inpatient encounter), then only the start date is tracked for the purpose of counting instances. A patient who begins at stay on January 1, ends that stay on January 3, begins another stay on January 10, and ends that stay on January 15, is considered to have two instances of the outcome.

Selecting Visit as an Instance will count any visit that includes the outcome as one instance, regardless of how many times it occurred. For instance, consider a patient administered an analgesic on each of the three days that make up an inpatient stay following some index event. If analgesic is an outcome, these three administrations will represent only one instance, because all three are associated with the same visit.

The output summary includes: Patients in Cohort (count of patients meeting query criteria); Patients with Outcome (of the patients in the cohort, count of patients that had the outcome in the time window); Mean (mean of the counts); Standard Deviation (standard deviation of the counts); Median (median of the counts); and Median (1+ instances) when patients with zero instances included in the analysis. In addition, T-Test statistics testing for the difference between the cohorts is included.

**Laboratory Results Analysis**

Lab Results can be included in the analysis only for the outcomes that are labs. Only the most recent lab values in the time window are included. For the lab results that are numeric, the outcome summary includes: Patients in Cohort (count of patients meeting query criteria); Patients with Outcome (of the patients in the cohort, count of patients that had the outcome in the time window); Mean (mean of the counts); and Standard Deviation (the standard deviation for lab values across patients in the cohort). In addition, T-Test statistics testing for the difference between the cohorts is included.

For the non-numeric lab results, three values are reported: counts of Negative; Positives; and Unknowns.

The counts are represented in the bar chart as percentages of the total counts.

**Outcome Definitions**

Table below outlines the definitions for each outcome. For outcome definitions consisting of more than one term, at least one term must match.

| **Surgical site infection / infection following procedure (90 days)** | | | | |
| --- | --- | --- | --- | --- |
|  | **Outcome definition** | | | |
|  |  | Diagnosis | ICD-10-CM T81.40 to T81.44, T81.49 | Surgical site infection or infection following a procedure |
| **Sepsis (90 days)** | | | | |
|  | **Outcome definition** | | | |
|  |  | Diagnosis | ICD-10-CM A40, A41, R65.2 | Sepsis |
| **Cellulitis of lower extremity (90 days)** | | | | |
|  | **Outcome definition** | | | |
|  |  | Diagnosis | ICD-10-CM L03.115, L03.116, L03.119 | Cellulitis of the lower extremity |
| **Wound dehiscence / disruption (90 days)** | | | | |
|  | **Outcome definition** | | | |
|  |  | Diagnosis | ICD-10-CM T81.3 | Wound dehiscence or disruption |
| **Mechanical complications (90 days, 1 and 2 years)** | | | | |
|  | **Outcome definition** | | | |
|  |  | Diagnosis | ICD-10-CM T84.0 group | Any of: broken or dislocated prosthesis, instability, mechanical loosening, periprosthetic osteolysis, or articular bearing surface wear |
| **Emergency department visit (90 days)** | | | | |
|  | **Outcome definition** | | | |
|  |  | Encounter | TriNetX Encounter Emergency | Emergency department visit |
| **Readmission (90 days)** | | | | |
|  | **Outcome definition** | | | |
|  |  | Encounter | TriNetX Encounter Inpatient | Readmission |
| **Periprosthetic fracture (1 and 2 years)** | | | | |
|  | **Outcome definition** | | | |
|  |  | Diagnosis | ICD-10-CM M97.0, M97.1 | Periprosthetic fracture |
| **Instability / dislocation (1 and 2 years)** | | | | |
|  | **Outcome definition** | | | |
|  |  | Diagnosis | ICD-10-CM T84.02 group | Instability or dislocation |
| **Reoperation (1 and 2 years)** | | | | |
|  | **Outcome definition** | | | |
|  |  | Procedure | CPT 27134, 27137, 27138, 27090, 27091, 27486, 27487, 27488 | Reoperation |
| **Periprosthetic joint infection (1 and 2 years)** | | | | |
|  | **Outcome definition** | | | |
|  |  | Diagnosis | ICD-10-CM T84.50 to T84.59 | Periprosthetic joint infection |

**Propensity Score Matching**

Cohorts were matched 1:1 using propensity score matching. Matching covariates included: age at index, sex, race, ethnicity, overweight and obesity (ICD-10 E66), hyperlipidemia (E78.5), type 2 diabetes mellitus (E11), long-term current use of insulin (Z79.4), chronic kidney disease (N18), nicotine dependence (F17), alcohol-related disorders (F10), and opioid-related disorders (F11).
